# Stature is an essential predictor of muscle strength in children

**DOI:** 10.1186/1471-2474-13-176

**Published:** 2012-09-18

**Authors:** Jean-Yves Hogrel, Valérie Decostre, Corinne Alberti, Aurélie Canal, Gwenn Ollivier, Emilie Josserand, Ilham Taouil, Dominique Simon

**Affiliations:** 1 Institut de Myologie, UPMC UM 76, INSERM U 974, CNRS UMR 7215, Paris Cedex 13, 75651, France; 2 AP-HP, Hôpital Robert Debré, Unité d’Epidémiologie Clinique, Paris, 75019, France; 3 Université Paris Diderot, Sorbonne Paris Cité, Paris, 75019, France; 4 Inserm, CIE 5, Paris, 75019, France; 5 AP-HP, Hôpital Robert Debré, Service d’Endocrinologie Pédiatrique, Paris, 75019, France

**Keywords:** Muscle strength, Dynamometry, Children, Growth retardation

## Abstract

**Background:**

Children with growth retardation or short stature generally present with lower strength than children of the same chronological age. The aim of the study was to establish if strength was dependent on variables related to stature in a population of healthy children and to propose practical predictive models for the muscle functions tested. A secondary aim was to test for any learning effects concerning strength measured at two successive visits by children.

**Methods:**

Hand grip, elbow flexion and extension, and knee flexion and extension were measured by fixed dynamometry in 96 healthy subjects (47 girls and 49 boys, aged from 5 to 17 years).

**Results:**

For the present paediatric population, muscle strength was highly dependent on height. Predictive models are proposed for the muscle functions tested. No learning effect between the first and the second visit was detected for any of the muscle functions tested.

**Conclusions:**

This work shows that strength measurements using fixed dynamometry are reliable in children when using appropriate standardization of operating procedures. It underlines the particular relationship between body stature and muscle strength. Predictive equations may help with assessing the neuromuscular involvement in children suffering from various disorders, particularly those affecting their stature.

## Background

The increasing number of therapeutic trials for neuromuscular diseases is generating a need for muscle strength reference values for healthy control children in order to evaluate the severity and the progression of patients' disease. Maximal voluntary isometric contraction (MVIC) has previously been measured to provide normative data for healthy children. This has generally involved the handgrip function, probably because its assessment in children is simple and straightforward [1-3]. For other muscle functions, handheld dynamometry [3-8] and isokinetic/isometric dynamometers [9,10] have been used. Surprisingly, quantitative muscle testing (QMT), also known as fixed dynamometry, which is a reliable and sensitive method, has rarely been used in healthy children [11] although it was the method chosen to assess primary or secondary criteria in several therapeutic trials (for instance, in Duchenne muscular dystrophy [12] and amyotrophic lateral sclerosis [13]). Several disorders are associated with delayed growth or short stature, which has rarely been considered when comparing the strength of children.

Actually, muscle strength norms are mostly expressed in relation to chronological age [14]. However, as pointed out by Rauch et al. [2], chronological age may be a poor variable to account for muscle strength because physical development, hence body size, is a substantial determinant of strength. To overcome the limited value of chronological age for reliably predicting strength, several predictive equations have been developed using additional variables, *e.g*. weight, height, body mass index (BMI) and sex [3,4,15]. Again, these predictive models mainly concern grip strength.

Even for other muscle functions, strength values are generally presented as linear forces in kg or N. However, the actions of flexor and extensor muscles around joints generally result in angular movements. Thus, it seems more appropriate to measure flexion and extension torques rather than linear forces. Torque is computed as the product between the length of the lever arm (the distance between the rotation axis and the application point of the force) and the force generated perpendicularly to this lever arm. In children, torque measurement is particularly relevant because lever arm length increases during growth [4].

The main aim of the study was to explore the possible relationships between the children stature and their ability to generate muscle strength expressed as torques (except for hand grip strength which is expressed in N) and to propose practical predictive models for the muscle functions tested, as already reported for healthy adults [16]. A secondary aim of the study was to test whether there was a learning effect concerning strength, as determined by measurements at two successive tests on children.

## Methods

### Participants

This study was open to healthy children of both sexes between 5 and 17 years of age. Exclusion criteria included muscle disease, treatment possibly influencing the neuromuscular system, practicing high-level sport (more than 5 hours a week in sports clubs) and occurrence of any illness or injury during the preceding month. Subjects were recruited from relatives of the hospital personnel, relatives of patient families, and advertisements displayed in hospitals and in the publications of various patient associations. Informed consent forms were signed by children and parents. This study received the approval of the Local Ethical Committee (CPP Ile-de-France IV).

### Auxological assessment

Height was measured to the nearest millimetre using a standard height gauge (SECA 216 Height Rod) and was also expressed in standard deviations (SD) with respect to French population references. Weight was measured to the nearest 0.1 kg using electronic scales (Tanita TBF-543).

### Strength measurement

Strength was assessed bilaterally for five muscle functions: handgrip, elbow flexion and extension, and knee flexion and extension. A QMT system was used for the measurements. This system was designed to measure force production in isometric conditions. It included a wall-mounted frame, a load cell, straps for attaching the load cell to the frame and the subject, a grip dynamometer, an examination table and a computer for feedback and force recording (details can be found at http://www.qmasystem.com). The subject was placed in standardized positions on the examination table and the examiner provided appropriate stabilizations during the efforts to avoid artefactual or compensatory movements.

Elbow flexion and extension strength were assessed in the supine position with the elbow at 90° flexion at the side of the trunk, the forearm in a neutral pro-supination position. The evaluator stabilized the subject’s upper limb by holding the anterior part of the shoulder with one hand and the lateral condyle of the elbow with the other hand.

Knee flexion and extension strengths were assessed in the sitting position, with hip and knee at 90° flexion. A flat cushion was installed below the distal part of the working thigh to ensure that the segment was horizontal. For knee extension, the evaluator placed one hand on the lateral part of the subject’s knee and the other hand on the proximal part of the thigh to prevent hip rotation and extension as compensatory movements. For knee flexion, the evaluator maintained the knee with both hands placed on the anterior distal part of the thigh.

For knee and elbow flexion and extension, the lever arm length was measured at each visit to compute the maximal torque produced around the joints. The strap was placed distally on the leg or the forearm segment with the distal edge of the strap at the level of the malleoli or the styloids, respectively. This length was measured as the distance between the rotation axis of the joint and the middle of the strap, to the nearest half-cm using a flexible measuring tape. The rotation axis of the knee was considered at the middle of the lateral part of the femoro-tibial interline, while the rotation axis of the elbow was taken at the epicondyle level.

Handgrip strength was measured while the subject was seated, the elbow at 90° flexion along the side of the trunk. The height of the examination table was adjusted so that the feet were flat on the floor with hips and knees each at a 90° angle. The contralateral hand of the subject was placed on the thigh. The grip handle width was adjusted to hand size. The evaluator supported the subject’s forearm and the device.

The test order was always the same: handgrip right and left, knee extension right and left, knee flexion right and left, elbow flexion right and left, and elbow extension right and left. The measurements were recorded by dedicated software (QMA computer software package).

Trials were carried out with verbal encouragement asking the subjects to provide maximal voluntary isometric contractions (MVIC) during about three seconds with one minute rest between trials. For each muscle function tested, if the difference between the first two measurements was below 10% of the higher value, that higher value was recorded. If not, measurements were repeated until two trials gave values with a difference lower than 10% of the higher value. The maximal value of two reproducible trials was recorded as the MVIC of the function. The force curves were visually checked to ensure that no overshoot or artefact was present.

Particular attention was given to making the subjects feel confident so as to help them to produce their true MVIC. Explanations were adapted to the maturity of the subject and were repeated until the child seemed to understand perfectly what was required of him/her. All measurements were performed by three examiners trained to the same operating procedures (standardized positioning procedures, lever arm measurements, verbal instructions, curve validation and reading…). Reliability between evaluators for both QMT and lever arm measurements was ensured before the study by a preliminary training demonstrating similar results obtained by the different evaluators on the same subjects. Statistical analyses were performed without adjustment on evaluators.

### Assessment of learning effects

To ensure that true MVIC was measured, a second measurement session was conducted. This second session allowed to test for learning effects, possibly arising from difficulties in understanding or shyness at first visit; it also allowed assessment of the reliability of the method. The second session took place between two days and three months after the first one.

### Data and statistical analysis

Quantitative variables are reported as medians (range), and qualitative variables as frequencies (percentages). The relationship between the various muscle functions was tested using Pearson’s correlation coefficient. Normality was assessed for all variables using the Kolmogorov-Smirnov statistics.

Knee and elbow flexion and extension are expressed as torque in newton.meters (N.m), and handgrip strength is expressed in newtons (N). The dominant hand side was defined as that with which the children wrote.

Paired t-tests were performed for each muscle function measured to detect any learning effect. Reliability was assessed by means of Bland-Altman plots and calculating intraclass correlation coefficients (ICC). Bland-Altman plots represent the differences between the strength values measured during the test and retest sessions against the means of these values. It shows the amount of disagreement between the two measures (via the differences) and how these differences are distributed. ICC_2,1_ was computed as a single-measure ICC with a two-way random effects model (absolute agreement). We considered coefficients of 0–0.20 as 'slight', 0.21-0.40 'fair', 0.41-0.60 'moderate', 0.61-0.80 'substantial' and >0.80 'almost perfect'. Standard errors of measurement (SEM) were computed as the SD of the differences between test and retest values divided by the square root of two. The SEM is a measure of absolute reliability and is expressed in the actual units. Relative SEM (%) was computed as absolute SEM divided by the mean of the measure.

Reference intervals for muscle functions were estimated by multiple linear regression. Age, height and sex were considered for model building. The SD was estimated as the standard deviation of the residual of the measurement of interest from regression on all parameters. The model fit was assessed by calculating the standard deviation scores (Z-score) as Z = (measurement – mean)/SD. The ordered Z-scores were plotted to provide a graphical check of normality using QQ-plot. The absence of heteroscedasticity was also checked by plotting Z-scores against height and age.

SPSS 15 (SPSS Inc., Chicago, IL) and SAS v9.2 (SAS Institute Inc, Cary, NC, USA) softwares were used for statistical analyses. The limit of statistical significance was set at an alpha risk of error of 0.05.

## Results

Ninety-six children (47 girls, 49 boys) were included in the study. The ages and anthropometric characteristics of this paediatric population are reported in Table 1. Eighty-seven children (90.6%) were right-handed and nine (9.4%) were left handed.

**Table 1:** Characteristics of the population, reported as medians (range)

	**Girls (n = 47)**	**Boys (n = 49)**
Age (years months)	10y 10mo (6y 2mo – 16y 7mo)	10y 8mo (5y 4mo – 16y 6mo)
Height (cm)	144.5 (111.7 – 170.3)	142.5 (109.5 – 181.0)
Height SD (cm)	0.66 (−1.88 – 2.14)	0.82 (−1.87 – 3.22)
Weight (kg)	34.9 (17.5 – 86.0)	35.7 (21.0 – 79.6)
BMI (kg/cm^2^)	17.1 (8.4 – 30.5)	17.7 (15.1 – 28.2)
BMI SD (kg/cm^2^)	0.05 (−1.52 – 2.70)	0.32 (−0.77 – 3.09)

**Figure 1:**
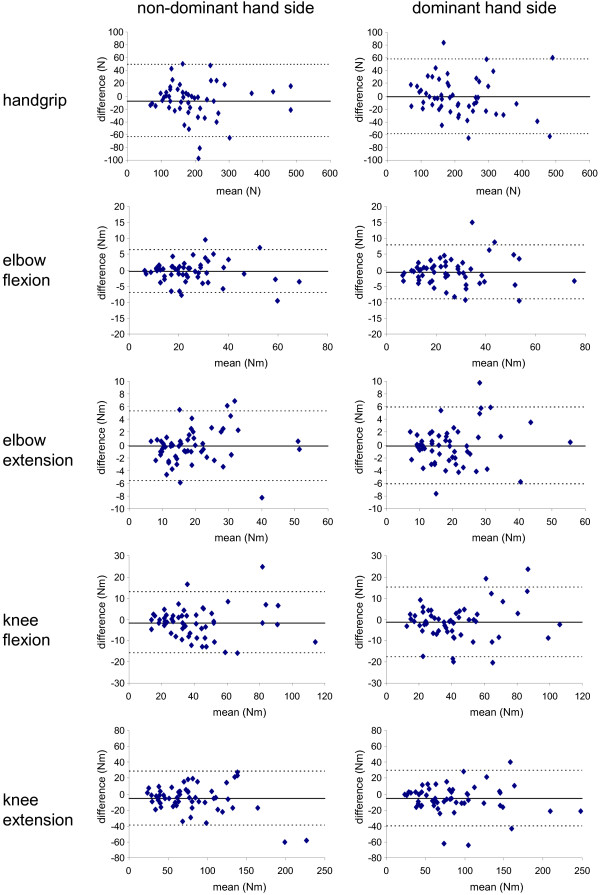
Bland-Altman plots for all muscle functions studied (n = 56 subjects).

Fifty-six children attended a second testing session to evaluate a possible learning effect. The median time between the two sessions was 42 days (2 – 85). No learning effect was detected: the differences between the results at the first and second visits were not significant for any of the muscle functions tested (all p > 0.05). Figure 1 presents Bland-Altman plots for each muscle function for both non-dominant and dominant hand sides. The differences between visit 1 and visit 2 plotted versus the mean torque for visit 1 and 2 were normally distributed and no trends were identified concerning the mean test and retest measures. The mean strength, the mean and SD of the differences between visit 1 and visit 2, the SEM, the relative SEM, the ICC and the 95%CI for ICC for each muscle function are shown in Table 2. The relative SEM was about 10 to 15% depending on the muscle function. As the results obtained during the two sessions were not significantly different, the values recorded during the first visit were used to compute the predictive equations.

**Table 2:** Test-retest reliability

**Muscle function**	**Side**	**Mean**	**Mean of difference**	**SD of difference**	**SEM**	**Relative SEM (%)**	**ICC**	**ICC 95%CI**
Handgrip	ND	196.1	−8.4	28.9	20.4	10.4	0.949	[0.911 ; 0.971]
	D	211.5	−1.2	29.6	20.9	9.9	0.957	[0.926 ; 0.975]
Elbow flexion	ND	24.2	−0.3	3.4	2.4	10.0	0.969	[0.947 ; 0.981]
	D	25.7	−0.6	4.2	3.0	11.7	0.954	[0.923 ; 0.973]
Elbow extension	ND	19.0	−0.2	2.7	1.9	10.2	0.962	[0.936 ; 0.977]
	D	19.2	−0.2	3.1	2.2	11.2	0.950	[0.916 ; 0.970]
Knee flexion	ND	40.7	−1.5	7.3	5.2	12.7	0.945	[0.908 ; 0.968]
	D	41.9	−1.4	8.3	5.8	14.0	0.930	[0.884 ; 0.958]
Knee extension	ND	78.7	−5.3	17.1	12.1	15.4	0.945	[0.908 ; 0.968]
	D	84.5	−6.0	17.6	12.4	14.7	0.926	[0.870 ; 0.957]

*ND*: non-dominant hand side; *D*: dominant hand side; *SD*: standard deviation; *SEM*: standard error of measurement; *ICC*: intraclass correlation coefficient; *CI*: confidence interval.

Data are provided in N for handgrip and Nm for the other muscle functions tested (n = 56 subjects).

The strength values for handgrip (p < 0.001), elbow flexion (p < 0.001), knee flexion (p = 0.044) and knee extension (p < 0.001) were all greater for the dominant than the contralateral side. This was not the case for elbow extension although a trend was observed (p = 0.245). The same was true for each sex separately, except for knee flexion for which only boys presented a greater strength on the dominant hand side (p = 0.020).

**Figure 2:**
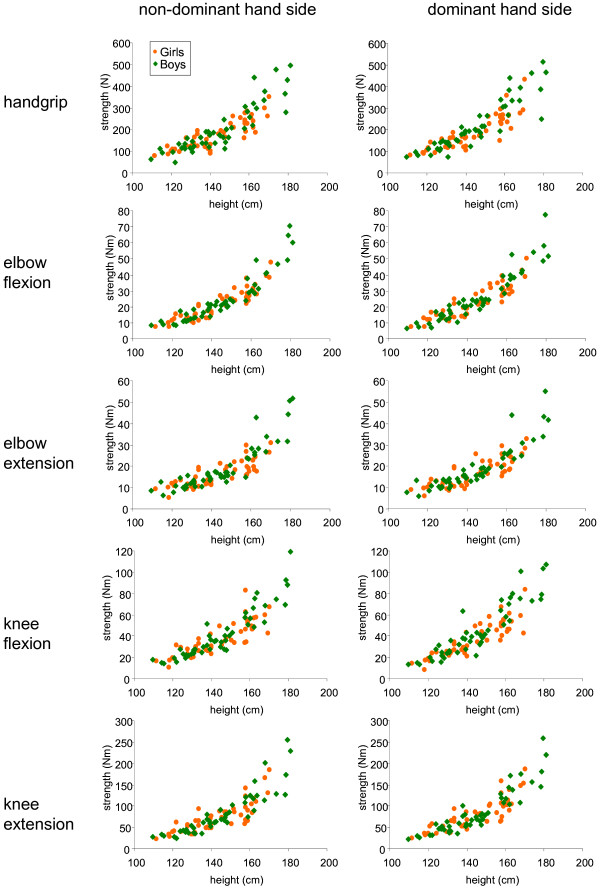
Muscle strength related to body height for boys and girls (n = 96 subjects).

The characteristics of the predictive model based on height, sex and age for the log transform of MVIC are given in Table 3 for each muscle function. As an alternative model, exponential regression may also be used to predict MVIC with height as the single explanatory variable (Table 4). Figure 2 presents the strength measured for the different muscle functions as a function of height, which was the most significant explanatory variable for strength as assessed by multiple linear regression. The strength for all muscle functions did not differ between girls and boys of the same height. Both types of model gave similar adjusted coefficients of determination (see Tables 3 and 4) although the Akaike information criteria were slightly better for the model based on height, sex and age.

**Table 3:** Equations predicting log (MVIC) using age, height and sex as variables (n = 96 subjects)

**Muscle function**	**Side**	**a**	**b**	**c**	**d**	**SD**_**log(MVIC)**_	**Adjusted R**^**2**^
		**(Intercept)**	**(Age coeff)**	**(Height coeff)**	**(Sex coeff)**		
Handgrip	ND	2.4555	0.0501	0.0152	−0.0727	0.2120	0.781
	D	2.2692	0.0416	0.0177	−0.1012	0.1925	0.826
Elbow flexion	ND	−0.5433	0.0298	0.0226	−0.0590	0.1603	0.894
	D	−0.5674	0.0317	0.0230	−0.0310	0.1840	0.870
Elbow extension	ND	0.0289	0.0319	0.0170	−0.0866	0.1950	0.791
	D	−0.0278	0.0321	0.0175	−0.0816	0.1893	0.807
Knee flexion	ND	0.4470	0.0372	0.0190	−0.0687	0.1935	0.830
	D	0.2679	0.0421	0.0203	−0.1368	0.2029	0.841
Knee extension	ND	0.4991	0.0182	0.0245	−0.0148	0.2251	0.809
	D	0.8783	0.0487	0.0199	−0.0239	0.2085	0.838

*ND*: non-dominant hand side; *D*: dominant hand side.

Handgrip strength is expressed in N; all other muscle functions are expressed in N.m. Age is expressed in years, height in cm, sex is 1 for girls and 0 for boys. SD_log(MVIC)_ is the residual standard deviation of the regression. The equation takes the form: log(MVIC) = a + b.age + c.height + d.sex, where a, b, c and d are the regression coefficients given in the table.

**Table 4:** Coefficients of equations predicting log(MVIC) using height as the sole variable (n = 56 subjects)

**Muscle function**	**Side**	**a**	**b**	**SD**	**Adjusted R**^**2**^
		**(Intercept)**	**(Height coeff)**		
Handgrip	ND	1.769	0.023	0.214	0.779
	D	1.692	0.024	0.193	0.825
Elbow flexion	ND	−0.975	0.028	0.172	0.883
	D	−1.014	0.028	0.192	0.864
Elbow extension	ND	−0.449	0.022	0.206	0.776
	D	−0.501	0.023	0.201	0.791
Knee flexion	ND	−0.104	0.025	0.201	0.824
	D	−0.338	0.028	0.218	0.823
Knee extension	ND	0.239	0.028	0.231	0.808
	D	0.203	0.028	0.218	0.830

*ND*: non-dominant hand side; *D*: dominant hand side.

Handgrip strength is expressed in N; all other muscle function are expressed in N.m. Height is expressed in cm. SD is the residual standard deviation of the regression. The equation takes the form: log(MVIC) = a + b.height, a and b being the regression coefficients given in the table. Knowing the height of the subject, this equation can be easily used by computing the theoretical strength as: MVIC = exp(a + b.height).

Correlations between the various muscle functions were highly significant (all p < 0.001) and were between 0.870 and 0.955.

## Discussion

The capacity of muscles to generate strength is one of the main features of maturation during child growth. We report paediatric strength values for quantified muscle testing with fixed myometry obtained from 96 children aged between 5 and 17 years. We also described equations for predicting strength of handgrip, elbow flexion and extension, and knee flexion and extension and observed that height is a major explanatory variable for muscle strength.

In previous studies, grip strength has been more extensively studied than other muscle functions. The strength values we report here are consistent with previous work in paediatrics for handgrip [2] and for other muscle functions tested by handheld dynamometry [4]. Child muscle strength has rarely been reported as torque, although torque is a more appropriate measure than linear force. Indeed, for the same torque, generated by the muscle during a maximal contraction for instance, the linear force depends on the length of the lever arm, defined as the distance between the application point of force and the rotation axis of the joint. Measuring the torque rather than linear force is therefore particularly important for longitudinal studies involving growing children.

The literature provides evidence of substantial inter-individual variability concerning strength expressed with respect to the age. A significant part of this variability may be due to differences in height among children in a given age class. Indeed, Niempoog et al. [15] recently indicated that “chronological age alone does not reflect the pubertal stage that leads to different physical performance”. Height was found to be strongly correlated to grip strength in several studies [15,17]. Our study suggests that height is a major explanatory variable also for strength of muscle functions other than handgrip and is in line with Parker et al. [18]. The strong correlations between the different muscle functions are a clue to a global effect of stature on the whole body muscle strength. We found that girls and boys cannot be overall distinguished according to strength when related to height, at least before 17 years of age. A strong relationship between muscle strength and height has previously been reported for pre-pubertal boys and girls [4,19] Also, the study of Newman et al. [20], implies that grip strength is similar in boys and girls under, approximately, 160 cm of height. Thus below 160 cm, norms for girls and boys appear to be the same when using height as the single explanatory variable. Sartorio et al. [21] observed that “gender differences disappeared when grip strength is normalized for fat free mass in children from 5 to 15”. Since height is closely correlated to lean body mass [22,23], it is not surprising that height explains a large part of the variability between individuals. Interestingly, the relationship between strength and height was accurately described by an exponential model, consistent with the observation that "lean body mass is an exponential function of height" [22].

The exponential model was also applied to linear force. Compared to torque-height relations, the mean adjusted R^2^ decreased from 0.825 ±0.035 down to 0.686 ±0.080 due to a larger inter-individual variability that can probably be explained by a lever arm effect.

We found that the dominant hand side was significantly stronger than non-dominant hand side for both sexes for handgrip, elbow flexion (in accordance for both sexes with Bäckman et al. [6]) and knee extension (only for boys in the same study). Concerning knee flexion, we found that only boys were stronger on the dominant hand side, in contrast with Bäckman et al. [6] who found this result only for girls. However, the effect of dominance for this muscle function was weak: in our study, the difference between the two sides was only 1 Nm for a mean strength of about 40 Nm.

Results did not differ between the test session and the retest session. It suggests that the QMT method can be used immediately, without preliminary training, for children in clinical trials. However, a habituation session may be useful to accustom children to the assessment procedures and make them feel confident with the evaluator.

ICCs are relative measures of reliability that have been used in many studies. They are generally good to excellent, as in the present study, particularly because the range of the measures is generally large. However, the assessment of reliability should not be limited to the use of ICC. Standard error of measurements (SEM) is a measure of agreement and serves as an index of absolute reliability; it has been much less widely used for evaluations of the performance of strength assessment techniques. The SEM for handgrip strength was about 20 N in our study, while Moleenar et al. [24] reported a SEM of about 11 N. The difference may be due to the larger age range, hence the larger range of strengths, of the children in our study. Indeed, when normalized to the mean of the measurements, the relative SEMs for the two studies are similar (10% and 9%, respectively). Meldrum et al. [25] used the same QMT measurement system with adults, and the standard error of the difference between test and retest reported can be used to compute the relative SEM: the relative SEM was between 3.9 and 12.8% in adults, to be compared to 9.8 to 15.1% for children in our study. This suggests lower reliability of strength measurement in children than adults possibly due to their poorer concentration or motivation through successive visits. However, strength measurement reliability has not yet been formally compared between children and adults.

As underlined by Jaric [26] in the field of sports medicine, "the primary goal of strength testing has often been to assess the objective value of muscle function independent of possible confounding factors". This is all the more true in the clinical field when the aim is to evaluate neuromuscular involvement in a disease. Moreover, when considering children, early or late maturation needs to be taken into account due to the direct link between body stature and muscle strength. This connection seems also to apply to adults as recently demonstrated by the close relationship between hand circumference, as an indicator of body stature, and grip strength [27].

In this study, we used a paediatric population aged from 5 to 17 years to develop models to predict handgrip strength and elbow and knee flexion and extension torque. Several authors have reported strong correlations between strength and height and have proposed models linking the two variables [2,17,19]. Similarly, Van den Beld et al. [3] observed that "height proved to be a better predictor for handgrip strength than age in children aged 4–11 years".

Our paediatric population counted a rather small number of children and was not necessarily representative of the general population of French children. Reliability was assessed by several indicators. First, there are limitations to using ICC, particularly its interpretation when the data include large inter-individual variability, which is the case for groups of children covering a wide range of growth/maturation stages. In such situations, ICC is only a rough indicator of reproducibility. Second, standard error of measurement (SEM) was used to quantify absolute agreement between test and retest values. Although our results indicate a satisfactory reproducibility between test and re-test results, some children were clearly less motivated during the second evaluation visit than the first. This behaviour could have led to an overestimation of the SEM. This also indicates that evaluation sessions for children should not be complicated, boring or long such that motivation and attention are maintained.

We report here strength values for muscle functions in particular protocol conditions (dynamometer type, body segment positions, number of attempts, maximal value scoring). The predictive equations proposed here are reliable only in the conditions specified. For informative comparisons, test conditions must be the same in patient populations to be assessed and in the normative control population. Note also that the strength values were established for isometric contractions and do not apply to dynamic (concentric or eccentric) contractions.

## Conclusions

This work provides strength values for several muscle functions in a paediatric population. It reveals the direct relationship between body height, hence physical maturation, and the strength generation capacity of children. Thus, as muscle strength depends on stature, chronological age should not be used as a single variable to predict normal strength. The predictive equations we report here could be used to evaluate muscle strength loss in children suffering from chronic disease, as possible growth retardation due to the disease can be taken into account. Short stature can be observed in diseases affecting directly body stature (genetic disorders, hormonal deficiency), in the case of prolonged pharmacological treatment such as glucocorticoid therapy or in the case of malnutrition or mistreatment. As a clinical application, the present work will help in assessing the effect of growth hormone on steroid myopathy in children with chronic diseases.

## Abbreviations

MVIC: Maximal voluntary isometric contraction; QMT: Quantitative Muscle Testing; ICC: Intraclass Correlation Coefficient; SEM: Standard error of measurement.

## Competing interests

The authors declare that they have no competing interest.

## Authors’ contributions

JYH designed the experiments, analysed the data and drafted the manuscript. VD, AC and GO performed the experiments and drafted the manuscript. CA and EJ analysed the data and drafted the manuscript. IT organized the experiments, analysed the data and drafted the manuscript. DS was the principal investigator of the study and designed the experiments, analysed the data and drafted the manuscript. All authors read and approved the final version of the manuscript.

## Pre-publication history

The pre-publication history for this paper can be accessed here:

http://www.biomedcentral.com/1471-2474/13/176/prepub
